# Effect of Hydrophobic and Hydrophilic Metal Oxide Nanoparticles on the Performance of Xanthan Gum Solutions for Heavy Oil Recovery

**DOI:** 10.3390/nano9010094

**Published:** 2019-01-12

**Authors:** Laura M. Corredor, Maen M. Husein, Brij B. Maini

**Affiliations:** Department of Chemical and Petroleum Engineering, University of Calgary, Calgary, AB T2N 1N4, Canada; laura.corredor@ucalgary.ca

**Keywords:** nanoparticle, metal oxide, polymer flooding, xanthan gum, heavy oil

## Abstract

Recent studies revealed higher polymer flooding performance upon adding metal oxide nanoparticles (NPs) to acrylamide-based polymers during heavy oil recovery. The current study considers the effect of TiO_2_, Al_2_O_3_, in-situ prepared Fe(OH)_3_ and surface-modified SiO_2_ NPs on the performance of xanthan gum (XG) solutions to enhance heavy oil recovery. Surface modification of the SiO_2_ NPs was achieved by chemical grafting with 3-(methacryloyloxy)propyl]trimethoxysilane (MPS) and octyltriethoxysilane (OTES). The nanopolymer sols were characterized by their rheological properties and ζ-potential measurements. The efficiency of the nanopolymer sols in displacing oil was assessed using a linear sand-pack at 25 °C and two salinities (0.3 wt % and 1.0 wt % NaCl). The ζ-potential measurements showed that the NP dispersions in deionized (DI) water are unstable, but their colloidal stability improved in presence of XG. The addition of unmodified and modified SiO_2_ NPs increased the viscosity of the XG solution at all salinities. However, the high XG adsorption onto the surface of Fe(OH)_3_, Al_2_O_3_, and TiO_2_ NPs reduced the viscosity of the XG solution. Also, the NPs increased the cumulative oil recovery between 3% and 9%, and between 1% and 5% at 0 wt % and 0.3 wt % NaCl, respectively. At 1.0 wt % NaCl, the NPs reduced oil recovery by XG solution between 5% and 12%, except for Fe(OH)_3_ and TiO_2_ NPs. These NPs increased the oil recovery between 2% and 3% by virtue of reduced polymer adsorption caused by the alkalinity of the Fe(OH)_3_ and TiO_2_ nanopolymer sols.

## 1. Introduction

Water-soluble polymers have been used in enhanced oil recovery (EOR) due to their ability to improve sweep efficiency by controlling water mobility, reducing water permeability in swept zones and contacting unswept zones of the reservoir [1]. Hydrolyzed polyacrylamide (HPAM) and xanthan gum are the most commonly used polymers for EOR [2]. Xanthan gum (XG) is a high molecular anionic polysaccharide produced by bacterium *Xanthomonas campestris* during the fermentation of a cellulosic backbone [3]. XG solution properties have been studied over the past 50 years. The main topics of interest include rheological behavior of XG solutions and their mixtures with other polymers [4,5,6,7,8,9], effect of temperature on polymer conformation [10,11,12], and effect of salinity on solution performance and polymer conformation [13,14].

In solution, XG exhibits two conformational states: an ordered helix conformation and a disordered coil conformation [13]. The conformation of the XG molecules depends on the ionic strength of the solution and the temperature. In the presence of salts, XG molecules adopt a rigid rod like structure (ordered conformation) because the negatively charged pyruvate molecules wrap around the backbone of the polymer [3,15]. In salt-free solutions, XG molecules have a highly extended and disordered conformation due to the electrostatic repulsion between their charged side chains and their backbone. At low temperatures, XG molecules adopt the helix structure, which converts to a disordered coil at high temperatures [3]. The temperature limit for XG was suggested to be between 70 °C and 90 °C by Wellington [16], Ryles [17], and Seright and Henrici [18]. These reports showed that XG solutions are thermally stable at salt concentrations where XG chains remained in an ordered structure.

At low concentrations, XG has been used as a stabilizer of NPs in aqueous solutions for thermal recovery of heavy oil [19] and for drilling fluids [20,21]. Other works include the study of the effect of NPs on the rheological behavior of XG solutions [22,23,24,25]. However, the role of NPs in modifying the polymer performance for oil recovery has not been widely studied. Recently reported studies have shown that the addition of silica NPs to polyacrylamide solutions increases their mobility control ability, temperature tolerance, and salt-tolerance [26,27,28,29,30,31,32,33,34,35] and, consequently, increases the oil recovery.

Saha and coworkers [36] investigated the effect of hydrophilic silica NPs on the stability of crude oil–XG emulsions. They reported that the addition of the silica NPs improved the stability of the emulsions at all polymer concentrations investigated (1000–5000 ppm). Nevertheless, higher emulsification and better stability periods were obtained at 5000 ppm of XG and 0.3 wt % of silica NPs. The higher emulsification was attributed to the adsorption of the NPs at the oil–water interface, which reduced the interfacial tension (IFT) and the oil droplet sizes and size distribution. Moreover, the NPs changed the wettability of the system from intermediately oil-wet to water-wet, improving the oil recovery between 18% and 20% at 30 and 80 °C, respectively.

Nanosilica particles have been the most-studied NPs for both nanoflooding and nanopolymer flooding processes. Other NPs, such as TiO_2_ and Al_2_O_3_, in aqueous solutions, have shown great potential to enhance oil recovery for both light and heavy oil [37,38,39,40]. To the best of the authors’ knowledge, there are no literature investigations on the effect of the addition of Fe(OH)_3_, TiO_2_, and Al_2_O_3_ NPs to XG solutions as a secondary recovery method. The primary objective of this study is to examine the feasibility of improving the performance of XG polymer flood through adding TiO_2,_ Al_2_O_3_, in situ Fe(OH)_3_, and surface-modified SiO_2_ NPs. Silica functionalization may increase the hydrophobicity of the NPs and, hence, enhance their dispersivity into the polymer solution. Silica functionalization may also tailor the interaction between the treated NPs and the polymer to improve the mobility control ability of the polymer solution and, consequently, increase the oil recovery.

## 2. Materials and Methods

### 2.1. Materials

The nanoparticles used in this study were fumed silica (99.5%, 7 nm), aluminum oxide (99.8%, 13 nm) (from Sigma-Aldrich, St. Louis, MO, USA), titanium dioxide (99.5%, between 10–30 nm) (from Skyspring nanomaterials, Houston, TX, USA), and in situ-prepared Fe(OH)_3_ NPs. Ferric chloride hexahydrate (FeCl_3_.6H_2_O, 98%) and sodium hydroxide, 5 M (From Sigma-Aldrich, St. Louis, MO, USA) were used to prepare the Fe(OH)_3_ NPs in situ. The silica NPs were modified with two silanes, triethoxy(octyl)silane (OTES, >97.5%) and 3-(methacryloyloxy)propyl]trimethoxysilane (MPS, 98%) (from Sigma-Aldrich, St. Louis, MO, USA). The solvents used for silica NP modification were ethanol (EtOH, 99%) and cyclohexane (99.5%) (from Fisher Scientific, Bartlesville, OK, USA). Xanthan gum (XG, MW> 2 x10^6^ Da) was supplied by MP Biomedicals (Santa Ana, CA, USA). Sodium dodecyl sulfate (SDS, ≥ 98.5%) (from Sigma-Aldrich, St. Louis, MO, USA) was the surfactant used in nanopolymer sol preparation, and formaldehyde solution (37 wt % in water, containing 10%–15% of methanol as a stabilizer) (from Sigma-Aldrich, St. Louis, MO, USA) was used as a biocide. The displacement tests were performed with silicon oil (2400 cP) supplied by Nye lubricants (Fairhaven, MA, USA). Brine was prepared using sodium chloride (NaCl, 99%). The silica sand (100–140 mesh) used for the displacement tests was supplied by AGSCO (Wheeling, IL, USA).

### 2.2. SiO_2_ NP Surface Modification

A mass of 4 g of SiO_2_ NPs dispersed into 80 mL of cyclohexane were mixed with 1.6 mL of MPS or 2.09 mL of OTES. The dispersion was stirred at 200 rpm for 12 h at room temperature for the reaction to take place. After treatment, the product was recovered by centrifugation at 2500 rpm for 30 min and washed three times with ethanol to remove the excess modifier. The precipitate was dried in an oven at 70 °C for 24 h. Masses of 3.44 g of SiO_2_-MPS and 3.29 g of SiO_2_-OTES NPs were recovered, since some NPs were lost during preparation.

### 2.3. Modified NP Characterization

Fourier-transform infrared (FTIR) spectra were recorded over the range of 4000–400 cm^−1^ in a FTIR spectrometer (model IRaffinity−1s, Shimadzu, Japan) using KBr for running the background spectrum. Thermogravimetric analysis (TGA), from 20 to 800 °C, were performed on Q600 TGA (TA instruments, Inc., New Castle, DE, USA) at a heating rate of 10 °C/min in air atmosphere.

### 2.4. Nanopolymer Sol Preparation

The NPs were first dispersed in DI water at 0.2 wt % and ultrasonicated for 1 h. SDS was added at 0.1 wt %, and the dispersions were stirred for 30 min. Then, XG at 0.4 wt % was added and stirred for 1 h. Finally, NaCl was added to the sample to achieve a concentration of 0.3 wt % or 1.0 wt %.

For the preparation of the nanopolymer sols with in situ Fe(OH)_3_ NPs, 0.2 g of the polymer and 0.05 g of SDS were dissolved into 35 mL of DI water. Then, 1.02 g of an aqueous solution of FeCl_3_.6H_2_O (19.98 g of FeCl_3_.6H_2_O in 25 mL of DI water) and 1.86 g of an aqueous solution of NaOH (5N) were added to the solution. The sample was left to mix at 150 rpm at 25 °C for 10 min. Then, 12.69 g of DI water were added. The reaction produces 0.3 wt % of NaCl, as a byproduct, according to the following reaction:

(R1) $$FeCl_{3\left(s\right)}+3NaOH_{\left(aq\right)}\;\to Fe{\left(OH\right)}_{3\left(s\right)}+3NaCl_{\left(aq\right)}.$$

### 2.5. Colloidal Stability and Particle Size

ζ-potential values of each nanopolymer sol were measured at 20 °C in absence of NaCl using a Zetasizer Nano ZS unit (Malvern Instruments Ltd., Malvern, UK), with uncertainty in the order of ±1% to ±6% of the reported value. The measurements were conducted after ultrasonicating each sample for 5 min. The ζ-potential values were measured at 0.2 wt % NaCl. These values could not be measured at higher ionic strength (0.3 wt % and 1.0 wt % NaCl) due to the high conductivity values (>5 mS/cm). Particle size measurements were carried out by dynamic light scattering (DLS) using a particle size analyzer (Zetasizer nano ZS) at 20 °C. The results are presented in Table S1 of the supplementary material. A digital pH meter (Fisher Scientific, model AB 15 plus, Santa Barbara, CA, USA) with an uncertainty of less than ±0.05 of the reported value was used to measure the pH values at 20 °C.

### 2.6. Viscosity of the Nanopolymer Sols

The viscosities of the nanopolymer sols were measured at 25 °C using a viscometer (Thermo Scientific™ HAAKE RotoVisco 1, Santa Barbara, CA, USA). The uncertainty of the reported valued remained between ±1% to ±7%. The shear rate was varied from 5.0 to 100 s^−1^.

### 2.7. Displacement Test in Linear Sand-Pack

A stainless-steel tube with 30.4 cm length and 2.54 cm i.d. was used as a holder for silica sand. The packing process was carried out by filling the holder with sand and frequently tapping it to ensure the sand was tightly packed. The volume of the sand grains was determined as the weight of the sand into the holder divided by the density of the grains (2.6 g/cm^3^). The pore volume (PV) of the porous media was calculated by subtracting the volume of sand grains in the holder from the total volume of the holder (154.4 cm^3^). Porosity was calculated as pore volume divided by total volume of the holder. For the displacement tests, the sand-pack is initially fully saturated with brine by evacuating the pore space with a vacuum pump and allowing the DI water to imbibe under high vacuum. In order to determine the permeability of the porous media (*K_abs_*) from Equation (1), DI water was injected at different flow rates (10, 20, 30, and 40 mL/min), and the corresponding pressure drop was recorded.

(1) $$K_{abs}=\frac{L}{A}\cdot \mu \cdot Q\cdot \frac{1}{\Delta P},$$

where *L* is the length of the pack, cm; *A* is the cross-sectional area of the pack, cm^2^; *µ* is the viscosity of the fluid, mPa.s; *Q* is the flow rate, cm^3^/s; and Δ*P* is the differential pressure across the sand-pack, atm.

The drainage process was carried out by injecting 2 pore volumes (PV) of silicon oil at 0.1 mL/min until the water fraction at the production end was less than 1%, and the pressure stabilized. The initial oil saturation was calculated by dividing the volume of produced water during oil flooding by the pore volume. After that, 1 PV of polymer solution or nanopolymer sol was injected followed by 2 PV of water. Cumulative oil recovery was calculated by dividing the sum of oil recovered from the chemical flood by the initial volume of oil in the sand-pack. The aqueous and oil phases were separated by heating the collected samples at 70 °C for 30 min in an oven.

## 3. Results and Discussion

### 3.1. FTIR and TGA Measurements of the Modified Silica NPs

**Figure 1:**
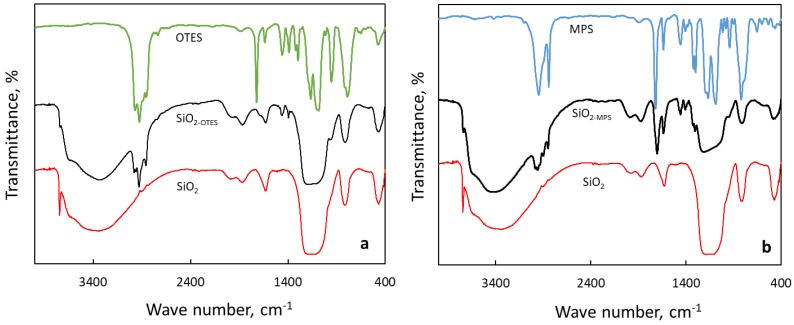
IR spectra of (**a**) SiO_2_, SiO_2_-OTES, and OTES, and (**b**) SiO_2_-MPS and MPS.

The FTIR spectra of unmodified and modified silica NPs after normalization of the peak area are shown in Figure 1. The asymmetric and symmetric stretching vibrations of C–H groups of MPS for the SiO_2_-MPS NPs were clearly observed around 2951 and 2481 cm^−1^, and the stretching vibration of C=O, methylene (=CH_2_), and vinyl (–CH=CH_2_) bending vibrations were observed at 1703, 1456, and 1406 cm^−1^ [41], respectively. For SiO_2_-OTES NPs, two peaks appear at 2926 cm^−1^ and 2858 cm^−1^ due to the intense symmetric and asymmetric stretching vibrations of the C–H bonds in the octyl groups [42]. A peak also appears at 1392 cm^−1^, which is assigned to the asymmetric deformation vibration of C–H bonds due to a slight substitution of the octyl groups in place of the –OH groups of the SiO_2_ NPs [43].

**Figure 2:**
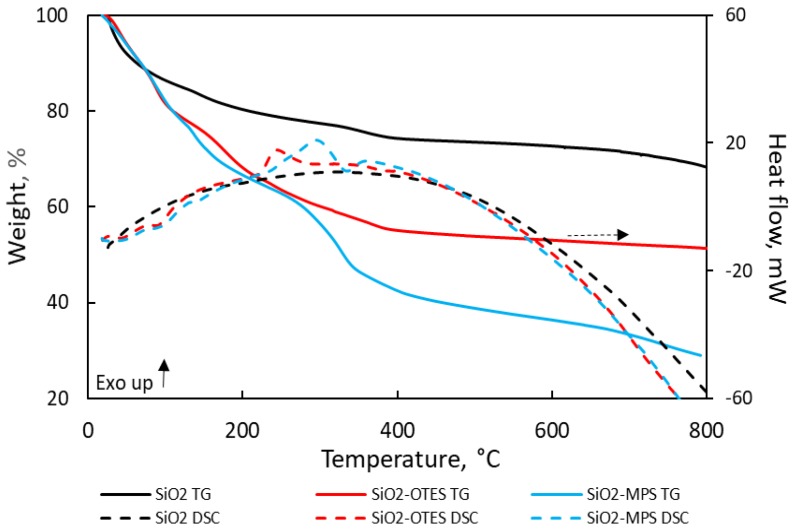
TG/DSC curves of SiO_2_-MPS and SiO_2_-OTES under air atmosphere.

The TGA thermograms shown in Figure 2 correspond to the unmodified and modified silica NPs. The thermogram of unmodified SiO_2_ NPs shows the desorption of physisorbed water up to 400 °C and the dehydroxylation of adjacent –OH groups between 400 and 800 °C [44]. The weight losses of each region were 25.71% and 6%, respectively. For modified silica NPs, the weight losses detected up to 100 °C were attributed to volatile solvents and unbound water. The weight loss for SiO_2_-OTES and SiO_2_-MPS in this region were 13.37% and 15.37%, respectively. The onset of oxidation of SiO_2_-OTES and SiO_2_-MPS was found at 200 °C. Temperatures for maximum oxidation were 245 °C for SiO_2_-OTES, and 300 °C for SiO_2-_MPS, and the weight loss was 15.48% and 23.41%, respectively.

### 3.2. Colloidal Stability

The colloidal stability of NP dispersions can be predicted by the magnitude of the ζ-potential. Dispersions with ζ-potential values greater than +30 mV or less than −30 mV typically have a high degree of stability [45]. NP dispersions with a low ζ-potential value will eventually agglomerate under the effect of interparticle attractions. Accordingly, most of the nanofluids (NPs dispersed in DI water) should potentially be unstable, as reported in Table 1. The mechanisms causing the instability of these NPs can be hydrophobic interactions among the modified silica NPs, or the hydrogen bonding between the silanols groups of the unmodified NPs.

It was observed that the unmodified silica nanofluid was stable despite its low ζ-potential value. The same observation was reported by Gun’ko et al. [46]. They suggest that the hydration layer formed between the silanols groups on the silica surface, and the water molecules through hydrogen bonding, prevent the agglomeration of the NPs. Effective steric hindrance leads to net repulsive interparticle forces despite the lack of charges on the silica surface [47]. The hydration effect was, nevertheless, not observed in the other hydrophilic NPs (i.e., Al_2_O_3_, TiO_2_, and Fe(OH)_3_). TGA analysis showed that the weight loss associated with the desorption of physisorbed water (up to 400 °C) for unmodified SiO_2_ NPs was 25.71%, while the weight losses for Al_2_O_3_, TiO_2_, and Fe(OH)_3_ were 0.49%, 3.78%, and 5%, respectively. The instability of Al_2_O_3_ and TiO_2_ in water was previously reported by Hendraningrat and Torsæter [38].

The ζ-potential for Al_2_O_3_ NPs in DI water is +16.4 mV at pH 6.75, which is relatively high, consistent with the fact that this pH is far from their isoelectric point (pH IEP = 9.1) [48]. When alumina is dispersed in water, it behaves like a basic oxide which consumes H^+^ ions. Thus, it possessed positive surface charges [48]. Upon adding SDS, the potential changes sign (−26.9 mV), which could be explained by the adsorption of SDS onto the surface of the positively charged NPs as a bilayer, with the sulfate headgroups exposed to the solution [49]. Formation of a surfactant bilayer with concomitant charge reversal of the alumina particles with anionic sodium dodecyl sulfonate surfactant was previously reported by Somasundaran and Fuerstenau [50]. In general, the addition of SDS increased the negative magnitude of the ζ-potential values of the NPs, but did not improve their stability.

The nanopolymer sols prepared in this study are stable (Table 1), mainly because of the stabilizing effect of the adsorbed polymeric chains onto the NPs surface inducing steric repulsion. In addition, the stability of the suspension increased due to the increase in the viscosity of the dispersion medium [51]. In general, polymer adsorption results from electrostatic and non-electrostatic interactions and the balance between these forces. The adsorption of XG chains onto the surface of the unmodified SiO_2_, Fe(OH)_3_, and TiO_2_ NPs likely occurs through hydrogen bonding between –OH groups on the surface of the NPs and the carboxylic groups on the side chains of the XG, and on the positively charged Al_2_O_3_ NPs through electrostatic attraction forces. The adsorption onto the modified SiO_2_-MPS and SiO_2_-OTES NPs likely occurs through hydrophobic interactions between the polymer backbone and the R–CH_3_ chain of the modifiers on the silica surface. It was observed that the addition of 0.2 wt % NaCl to the nanopolymer sols reduced their ζ-potential values, but did not destabilize them.

### 3.3. Viscosity Measurements

**Figure 3:**
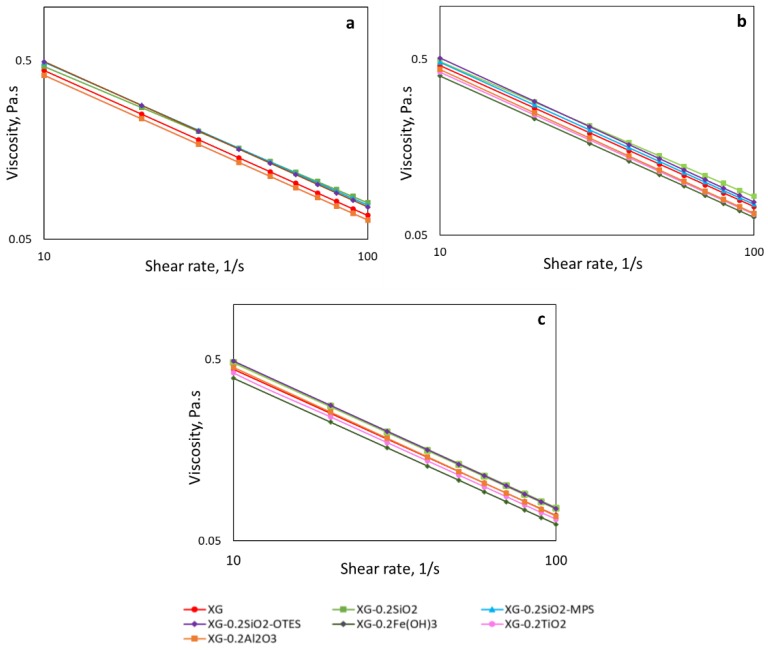
Viscosity of 4000 ppm XG solutions with 1000 ppm SDS and SiO_2_, SiO_2_-MPS, SiO_2_-OTES, Al_2_O_3_, TiO_2_, and Fe(OH)_3_ at **a**) 0 wt %, (**b**) 0.3 wt %, and (**c**) 1.0 wt % NaCl concentration at 25 °C.

The XG polymer solutions and nanopolymer sols exhibited shear-thinning behavior, which is attributed to the uncoiling and partial alignment of the XG chains at the high shear rate region (Figure 3a). The 4000 ppm XG solutions with 0.3 wt % and 1 wt % salt had slightly higher viscosity than the salt-free solution. This behavior was previously reported by Wyatt and Liberatore [13,52]. They found that, below a critical polymer concentration (C_c_ ~2000 ppm), the viscosity decreases upon addition of salt. The addition of counterions (Na^+^) contributes to neutralizing the electrostatic interactions between charges along the backbone of the XG chain, which allows the chain to fold, causing a reduction in the hydrodynamic size of the macromolecules. Since the XG chains decrease in size, the number of interactions with neighboring chains also decreases. For polymer concentrations >C_c_, the interaction among the XG chains increases, and its effect on the viscosity of the solution becomes more important than the negative effect caused by the decrease in the hydrodynamic size of the macromolecules.

The addition of unmodified silica, SiO_2_-MPS, and SiO_2_-OTES NPs to the XG–SDS solutions has a positive effect on the viscosity values at all salt concentrations (Figure 3). For the salt-free nanopolymer sols, the increment in viscosity can be attributed to the interaction between –OH groups on the surface of the NPs (modified and unmodified) with the carboxylic groups on the trisaccharide side chains of the XG through hydrogen bonding [25] and hydrophobic interactions between the modifiers and the backbone of the polymer. Then, the unmodified and modified silica NPs act as physical crosslinker between polymer chains. When NaCl is added to these nanopolymer sols, the hydrophobic interactions between SDS micelles, NPs, and the XG chains increases, due to the screening of the electrostatic repulsion between them [53,54]. The repulsion forces between the attached SDS micelles and SDS-coated NPs will prevent the coiling of the XG chains, facilitating the formation of a tridimensional network with other NP–XG–SDS complexes, increasing the viscosity.

The reduction in viscosity of XG–SDS solution caused by the addition of Fe(OH)_3_, Al_2_O_3_, and TiO_2_ NPs suggests high adsorption of polymer molecules at the NP surface. High polymer adsorption on the NP surface reduces the viscosity of the polymer solution because it reduces the polymer concentration in the liquid phase and diminishes the bridging of polymer molecules with the NPs. The reduction in the polymer viscosity was observed at all salinities.

The viscosity data of XG polymer solutions and nanopolymer sols exhibits a good fit to Ostwald–de Waele model (Equation 2). Nevertheless, a previous model developed by the authors [55] and based on multilayer perceptron (MLP) neural network can also be used for predicting the viscosity of nanopolymer sols. The rheological parameters (n and K) are presented in Table 2).

(2) $$µ=K\cdot {\dot{\gamma }}^{n-1},$$

where n is the flow index, dimensionless, and *K* is the consistency factor, Pa.s^n^.

### 3.4. Displacement Test in Linear Sand-Pack

**Figure 4:**
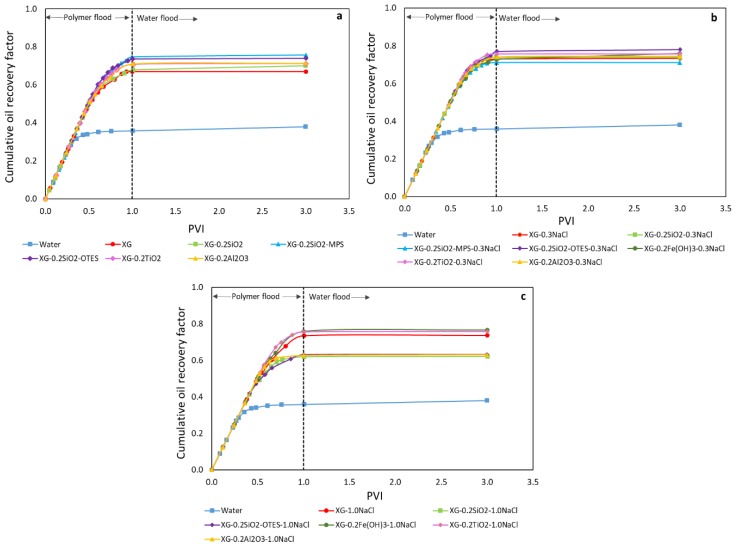
Cumulative oil recovery of 4000 ppm XG solutions with 1000 ppm SDS and SiO_2_, SiO_2_-MPS, SiO_2_-OTES, Al_2_O_3_, TiO_2_, and Fe(OH)_3_ at a) 0 wt %, (**b**) 0.3 wt %, and (**c**) 1.0 wt % NaCl concentration at 25 °C.

Table 3 presents the absolute permeabilities and porosities of the sand-packs used in this study and Figure 4 shows the cumulative oil recovery curves for water, polymer, and nanopolymer sols floods. The *r*^2^ values of the Δ*P*/L vs. Q/A curves used to calculate the absolute permeabilities were between 0.9970 and 0.9999. The cumulative oil recovery for waterflooding (WF) and polymer flooding was 38% and 67%, respectively. The addition of NPs to the salt-free XG solution increased its cumulative oil recovery between 3% and 9%. The increment on oil recovery is attributed to the improvement of the injected fluid and oil mobility ratio. The highest oil recovery was obtained with MPS- and OTES-modified SiO_2_ NPs.

At 0.3 wt% NaCl, NPs increased the cumulative oil recovery between 1% and 5%, except for SiO_2_-MPS. The oil recovery of SiO_2_-MPS was 2% lower than that of the XG solution. The reduction of the incremental oil recovery showed by all NPs can be attributed to NP and polymer adsorption on the sand grains induced by the addition of NaCl. Since the nanopolymer sols have a pH higher than 2 (between 6.04 and 10.62), the sand grain surface is likely negatively charged. Then, the formation and adsorption of NPs aggregates, and the adsorption of XG chains on the surface of the sand grains [56,57] is promoted by the counterions (Na^+^)_._ The Δ*P* values during nanopolymer flooding increased as salinity increased, due to the permeability reduction caused by the adsorption of NPs aggregates and XG chains within the porous medium (Table 3). The highest Δ*P* value during nanopolymer flooding was obtained by the injection of SiO_2_-MPS nanopolymer sol, which is in agreement with its low cumulative oil recovery. The highest oil recovery was obtained with SiO_2_-OTES NPs, which showed higher increment on XG viscosity and lower Δ*P* values.

At 1.0 wt % NaCl, the addition unmodified SiO_2_, SiO_2_-MPS, SiO_2_-OTES, and Al_2_O_3_ reduced the cumulative oil recovery between 5% and 12%. However, the addition of Fe(OH)_3_ and TiO_2_ NPs increased the cumulative oil recovery between 2% and 3%. The performance of the Fe(OH)_3_ and TiO_2_ NPs can be attributed to lower polymer adsorption on the surface of the sand grains. The alkaline nature of both nanopolymer sols (Fe(OH)_3_ pH = 10.62, and TiO_2_ pH = 8.15) increases the negative charge density of the sand grains [57] and the negative charges on the XG chains through the deprotonation of the carboxylic acids [12], thus, the repulsive forces between the XG chains and the sand grains increases, which leads to lower adsorption of the polymer. The high ΔP values during the injection of Fe(OH)_3_ and TiO_2_ nanopolymer sols are related mainly to the adsorption of NPs aggregates on the sand grains. In general, Fe(OH)_3_ and TiO_2_ nanopolymer sols were the only nanopolymer sols that show the same incremental oil recovery at 0.3 wt % and 1.0 wt % NaCl. It seems that the increment of the repulsive forces between the XG chains and the sand grains can mitigate the negative effect caused by the NP adsorption on the sand grains.

## 4. Conclusions

In this study, formulation and characterization of nanopolymer sols of XG with hydrophobic and hydrophilic metal oxide NPs is reported. The properties of the nanopolymer sols were explored using ζ-potential and viscosimetry. The performance of all nanopolymer sols was evaluated by conducting heavy oil recovery tests in linear sand-packs. The role of the physical and chemical interactions between NPs and XG solutions, and the relationship between their hybrid structure, properties, and their performance were investigated.

The colloidal stability of the NPs dispersed in DI water and XG solution was evaluated by the ζ-potential measurements. The NP dispersions in deionized (DI) water exhibited low ζ-potential values, which suggested low dispersion stability. Nevertheless, the addition of XG induced steric stabilization. It was observed that the addition of untreated silica, SiO_2_-MPS, and SiO_2_-OTES NPs improved the thickening behavior of the XG solution. However, Fe(OH)_3_, Al_2_O_3_, and TiO_2_ NPs decreased the viscosity of the polymer solution. The displacement tests showed that the breakthrough time occurred later with the injection of the nanopolymer sols, since the reduction of mobility ratio increased the macroscopic efficiency. At 0 wt % and 0.3 wt % NaCl, the addition of NPs increased the cumulative oil recovery between 3% and 9%, and between 1% and 5%, respectively. At 1.0 wt % NaCl, unmodified SiO_2_, SiO_2_-MPS, SiO_2_-OTES, and Al_2_O_3_ reduced the cumulative oil recovery between 5% and 12%, whereas Fe(OH)_3_ and TiO_2_ NPs increased it between 2% and 3%.

## Figures and Tables

**Table 1:** ζ-potential and pH values of xanthan gum (XG) nanopolymer sols at 20 °C.

		NaCl Concentration, wt %					
		0		0.2		0.3	1
NP Type	Dispersion Medium	ζ-Potential	pH	ζ-Potential	pH	pH	pH
SiO_2_	DI water	−17.5	6.89	-	-	-	-
	DI water–SDS	−25.1	6.73	-	-	-	-
	XG–SDS	−65.7	6.62	−56.3	6.44	6.73	6.89
SiO_2_-OTES	DI water	−20.7	7.01	-	-	-	-
	DI water–SDS	−28.3	6.89	-	-	-	-
	XG–SDS	−65.9	6.55	−57.8	6.38	6.60	6.75
SiO_2_-MPS	DI water	−20.3	7.06	-	-	-	-
	DI water–SDS	−29.4	6.77	-	-	-	-
	XG–SDS	−65.7	6.35	−59.1	6.22	6.61	6.85
TiO_2_	DI water	−25.3	6.95	-	-	-	-
	DI water–SDS	−28.8	6.78	-	-	-	-
	XG–SDS	−53.8	7.04	−46.4	6.87	7.41	8.15
Al_2_O_3_	DI water	16.4	6.75	-	-	-	-
	DI water–SDS	−26.9	6.54	-	-	-	-
	XG–SDS	−67.7	6.32	−59.5	6.04	6.48	6.64
Fe(OH)_3_	XG	-	-	-	-	10.5	10.62

**Table 2:** Power law parameters for nanopolymer sols.

Sample	K, Pa.s^n^	n	*r* ^2^
XG	2.8601	0.188	0.9972
XG–0.3NaCl	2.7642	0.199	0.9986
XG–1.0NaCl	2.8308	0.193	0.9982
XG–0.2SiO_2_	2.6939	0.237	0.9903
XG–0.2SiO_2_–0.3NaCl	2.8466	0.212	0.9989
XG–0.2SiO_2_–1.0NaCl	3.0761	0.195	0.9989
XG–0.2SiO_2_–OTES	3.2163	0.186	0.9988
XG–0.2SiO_2_–OTES–0.3NaCl	3.3465	0.181	0.9987
XG–0.2SiO_2_–OTES–1.0NaCl	3.1808	0.187	0.9991
XG–0.2SiO_2_–MPS	3.1175	0.198	0.9991
XG–0.2SiO_2_–MPS–0.3NaCl	3.0886	0.193	0.9991
XG–0.2SiO_2_–MPS–1.0NaCl	2.8506	0.186	0.9991
XG–0.2Fe(OH)_3–_0.3NaCl	2.5509	0.197	0.9992
XG–0.2Fe(OH)_3–_1.0NaCl	2.5132	0.195	0.9992
XG–0.2Al2O_3_	2.6541	0.189	0.9985
XG–0.2Al2O_3_–0.3NaCl	2.8708	0.182	0.9989
XG–0.2Al2O_3_–1.0NaCl	2.9945	0.179	0.9989
XG–0.2TiO_2_	2.6801	0.19	0.9996
XG–0.2TiO_2_–0.3NaCl	2.7115	0.192	0.9993
XG–0.2TiO_2_–1.0NaCl	2.9036	0.232	0.9993

**Table 3:** Properties of the sand-pack and Δ*P* after water and polymer flooding.

Experiment	*K_abs_*, D	Ф	Δ*P* after Polymer Flooding, psi/ft	Δ*P* after Waterflooding, psi/ft
Water	6.25	0.35	-	1.9
XG	5.93	0.31	8.2	4.5
XG–0.3NaCl	6.49	0.34	35.7	5.0
XG–1.0NaCl	5.99	0.34	120.0	10.1
XG–0.2SiO_2_	5.65	0.33	156.0	17.4
XG–0.2SiO_2_–0.3NaCl	5.96	0.34	161.0	25.4
XG–0.2SiO_2_–1.0NaCl	6.56	0.36	202.0	35.2
XG–0.2SiO_2_–OTES	6.09	0.35	82.0	26.0
XG–0.2SiO_2_–OTES–0.3NaCl	6.66	0.36	92.0	30.6
XG–0.2SiO_2_–OTES–1.0NaCl	5.64	0.34	185.0	32.0
XG–0.2SiO_2_–MPS	5.78	0.33	98.0	22.0
XG–0.2SiO_2_–MPS–0.3NaCl	6.49	0.36	173.0	43.4
XG–0.2SiO_2_–MPS–1.0NaCl	6.06	0.35	196.0	57.0
XG–0.2Al_2_O_3_	6.16	0.35	127.5	40.0
XG–0.2Al_2_O_3_–0.3NaCl	6.52	0.35	136.1	53.5
XG–0.2Al_2_O_3_–1.0NaCl	6.77	0.37	230.9	65.9
XG–0.2TiO_2_	5.96	0.32	56.0	24.0
XG–0.2TiO_2_–0.3NaCl	6.02	0.33	99.0	27.1
XG–0.2TiO_2_–1.0NaCl	6.67	0.36	136.9	37.5
XG–0.2Fe(OH)_3_–0.3NaCl	5.78	0.31	150.0	65.0
XG–0.2Fe(OH)_3_–1.0NaCl	5.94	0.32	154.0	88.0

## Supplementary Materials

The following are available online at http://www.mdpi.com/2079-4991/9/1/94/s1, Table S1: Average particle size measurement using DLS.

## Abbreviations

The following abbreviations are used in this manuscript:

A	cross-sectional area of the pack
D	darcy
FeCl_3_.6H_2_O	iron(III) chloride hexahydrate
HPAM	hydrolyzed polyacrylamide
IFT	interfacial tension
K	permeability
*K_abs_*	absolute permeability
*K*	consistency factor
KBr	potassium bromide
*L*	length of the pack
MW	molecular weight
n	flow index
PV	pore volume
PVI	pore volume injected
*Q*	volumetric flow rate
SDS	sodium dodecyl sulfate
SiO_2_-MPS	silica modified with 3-(methacryloyloxy)propyl]trimethoxysilane
SiO_2_-OTES	silica modified with octyltriethoxysilane
WF	waterflooding
XG	xanthan gum
Δ*P*	differential pressure across the sand-pack
Φ	porosity
$\dot{\gamma }$	shear rate
µ	viscosity of water
