# Supplementary material for: Effect of Hydrophobic and Hydrophilic Metal Oxide Nanoparticles on the Performance of Xanthan Gum Solutions for Heavy Oil Recovery
Source: Nanomaterials (Basel). 2019 Jan 12;9(1):94. doi: 10.3390/nano9010094 (PMC6358868; doi:10.3390/nano9010094)
Supplement: Supplementary file 1 [file nanomaterials-09-00094-s001.pdf]

Table S1. Average particle size measurement using DLS.

Nanoparticle type	NaCl concentration, wt.%	Particle size			
		size, nm	% intensity	size, nm	% intensity
SiO_2	0	29.7	80.6	3198.7	39.4
	0.3	47.4	77.5	3345.1	22.5
	1.0	53.1	65.3	3640.7	34.7
SiO_2-OTES	0	28.5	87.5	3320.4	12.5
	0.3	55.3	81.2	3287.9	18.8
	1.0	61.3	77.5	3647.7	22.5
SiO_2-MPS	0	76.0	77.2	3188.4	22.8
	0.3	89.7	50.7	3870.4	49.3
$Fe(OH)_3$	0.3	16.7	80.4	3032.2	19.6
	1.0	26.5	67.9	3156.8	32.1
TiO_2	0	55.0	81.3	3134.2	18.7
	0.3	58.4	74.5	3361.3	25.5
	1.0	66.4	69.0	3583.4	21.0
Al_2O_3	0	69.8	87.9	3325.6	12.1
	0.3	80.0	54.1	3525.3	45.9
	1.0	87.9	51.7	3897.1	48.3
